# Related Health Factors of Psychological Distress During the COVID-19 Pandemic in Spain

**DOI:** 10.3390/ijerph17113947

**Published:** 2020-06-02

**Authors:** Juan Gómez-Salgado, Montserrat Andrés-Villas, Sara Domínguez-Salas, Diego Díaz-Milanés, Carlos Ruiz-Frutos

**Affiliations:** 1 Department of Sociology, Social Work and Public Health, Faculty of Labour Sciences, University of Huelva, 21007 Huelva, Spain; salgado@uhu.es (J.G.-S.); frutos@uhu.es (C.R.-F.); 2 Safety and Health Postgraduate Programme, Universidad Espíritu Santo, Guayaquil 092301, Ecuador; 3 Department of Social, Developmental and Educational Psychology, Faculty of Education, Psychology, and Sports Sciences, University of Huelva, 21007 Huelva, Spain; montserrat.andres@dpsi.uhu.es; 4 Department of Psychology, Universidad Loyola Andalucía, 41704 Dos Hermanas, Sevilla, Spain

**Keywords:** COVID-19, psychological distress, pandemic, quarantine, mental health, public health, risk assessment

## Abstract

Measures to prevent and contain the COVID-19 health crisis include population confinement, with the consequent isolation and interruption of their usual activities. The aim of the study is to analyse psychological distress during the COVID-19 pandemic. For this, a cross-sectional observational study with a sample of 4180 people over the age of 18 during quarantine was developed. Variables considered were sociodemographic variables, physical symptoms, health conditions, COVID-19 contact history and psychological adjustment. The data were collected through a self-developed questionnaire and the General Health Questionnaire (GHQ-12). Bivariate analyses were performed, including Chi-Squared test and Student’s T-test. Predictive ability was calculated through logistic regression. Results obtained showed a high level of psychological distress (72.0%), with a higher percentage in women and people of lower middle age. Statistically significant differences were found in the variable working situation (χ² = 63.139, *p* *≤* 0.001, V = 0.123) and living with children under the age of 16 (χ² = 7.393, *p* = 0.007, V = 0.042). The predictive variables with the highest weight were sex (OR = 1.952, 95% IC = (1.667, 2.286)), presence of symptoms (OR = 1.130, 95% CI = (1.074, 1.190)), and having had close contact with an individual with confirmed COVID-19 (OR = 1.241, 95% CI = (1.026, 1.500)). These results could enrich prevention interventions in public health and, in particular, in mental health in similar pandemic situations.

## 1. Introduction

Coronavirus Disease 19 (COVID-19) has created a rapidly widening health crisis with dramatic consequences. On 31 December 2019, the Wuhan Municipal Health Commission in China notified the World Health Organization (WHO) of 27 cases of pneumonia of unknown origin [1]. On 30 January 2020, WHO declared an international Public Health emergency following the COVID-19 outbreak that began in Wuhan, China. By that date, 83 cases had been identified across 18 different countries outside China [2,3].

Following the increase in the spread to more than 118,000 cases in 114 countries and 4291 deaths, on 11 March 2020, WHO reported its pandemic consideration [4]. As a result of the rapid evolution of the public health emergency situation caused by COVID-19 and to prevent the containment of the virus and mitigating the health, social and economic impact, in Spain, the state of alarm was declared on 14 March 2020 [5]. Extraordinary temporary protection measures were put in place to ensure the safety of citizens. These measures involve the limitation of free movement of persons; the suspension of face-to-face educational activity; commercial activity except that relating to goods of first need; cultural, leisure and sporting activities; cult activities and civil and religious ceremonies, including funerals; and, in general, any activity involving crowds of people. Regarding the maintained activities, the need for people to keep at least one metre away from each other was emphasised [5]. Subsequently, on 29 March 2020 [6], measures were tightened to promote the control of the epidemic, limiting mobility to the maximum, except for tasks related to essential services that allow the country’s minimal functioning.

As Holmes et al. have reported, the coronavirus pandemic is causing a profound impact on the population with physical and psychological consequences [7]. The psychological distress associated with the pandemic health crisis has already been studied and it is estimated that 38.2% of the European population suffers from a mental disorder. [8]. Data from studies conducted in previous epidemics of Severe Acute Respiratory Syndrome (SARS), Middle East Respiratory Syndrome (MERS), influenza A virus subtype H1N1, Ebola, or initiation of COVID-19 show that quarantine as a protective measure has psychological effects on the population [9,10,11]. Quarantine has been linked to the presence of high depressive symptomatology [11,12,13], anxiety [14,15], stress [16], post-traumatic stress [13,17,18,19], anger [14], or psychological distress [20] mainly. Results from studies regarding psychological stress due to quarantine as a preventive measure of pandemia, vary form 24.6% and 56.0% [20,21,22,23,24]. Regarding the individual preventive measures, previous studies have identified that implementing more preventive and social avoidance measures relates to a higher level of anxiety [25,26,27].

On the other hand, the risk factors associated with greater distress have been identified, such as having adequate supply (of food or goods of first need), quarantine [20], lower level of training [21], lower level of health perception [23], degree of risk control, and the perception of risk [20]. In the opposite direction, the main protective factors identified associated with reduced psychiatric morbidity have been taking personal prevention/protection and clothing disinfection measures [20], clear communication of directives, and precautionary measures [24]. As for sex, the results are contradictory. While some studies suggest that women are at increased risk of psychological distress [21,28,29], others find that the risk is higher in men [20]. Studies focused on assessing the impact of confinement on anxiety, depression or post-traumatic stress have also identified other related factors such as the presence of disease symptoms [16], the level of health perception [16,23], the duration of the quarantine, and the degree of compliance with the disease [30].

Studies on psychological distress caused by the COVID-19 pandemic in the population are scarce. In China, a study conducted at the beginning of the quarantine revealed that 58.3% of participants were psychologically involved. Accurate and up-to-date information, especially related to preventive measures, was associated with lower levels of anxiety, depression, and stress [31]. Another study that analysed the contents on social networks before and after the COVID-19 pandemic identified an increase in the expression of negative emotions and social risk, and a decrease in the expression of positive feelings and satisfaction with life [32]. Qiu et al. identified greater psychological distress during the COVID-19 pandemic in women, population over 60 and with higher educational level [33]. In Iran, the level of psychological distress among the population was significantly higher than that of the Chinese population. In addition, the protective factors identified varied, including working from home, among others [34]. On the other hand, the level of psychological distress described in Brazil during the COVID-19 health crisis was moderately and contradictorily higher among the younger population [35]. As for the Italian population, psychological distress was identified as high and very high, and the identified risk factors were having a history of stressful situations or medical problems, having an infected acquaintance or family member, working away from home, or not having children, among others [36]. Other authors identified that people with a confident attachment style or uncomfortable with closeness are less likely to develop psychological distress during the quarantine [37].

In Spain, being female, younger, having negative self-perceptions about aging, being more time exposed to news about COVID-19, having more contact with relatives different to those with whom they co-reside, fewer positive emotions, less perceived self-efficacy, lower quality of sleep, the higher expression of emotion and higher loneliness have been associated with psychological distress during isolation measures as a result of the COVID crisis [38].

This variability in the risk and protective factors of psychological distress against COVID-19 suggests the need for further epidemiological studies to consolidate the results. Although there is agreement on the increase of psychological distress in the population during the pandemic, the characteristics of this situation are disparate, and the phenomenon is not yet clearly described [39].

The objective of the study is to analyse the psychological distress in a Spanish population sample during the COVID-19 pandemic, identifying the predictive character and role that sociodemographic variables, the presence of physical symptoms, and other health-related variables may have. As a hypothesis, it is stated that the health crisis caused by COVID-19 does not generate psychological distress in the population.

## 2. Materials and Methods

This research employed a cross-sectional observational study design.

### 2.1. Participants

The initial sample consisted of 4615 people, recruited between March 26 (13 days after the start of confinement) and April 26. As inclusion criteria for the participants, the following were established: (i) being 18 years of age or older, (ii) living in Spain during the COVID-19 pandemic, and (iii) accepting the informed consent.

435 questionnaires were eliminated for having a percentage of questions answered of less than 99%, which resulted in a final sample of 4180 participants distributed in 50 provinces and the two small Spanish autonomous cities located in North Africa.

Based on the sociodemographic characteristics of the sample, 74.0% were women and 26.0% men. The distribution by sex of the Spanish general population is 49.0% women and 51.0% men [40]. The average age of the participants stood at 40.26 years (SD = 13.18). The most common marital status was married or living as a couple (57.8%) followed by being single (33.9%). Most of the participants had university studies or higher educational level (76.9%) and the 20.0% had upper secondary education. Demographic data of the general Spanish population indicate that 17% of the population has university studies [40]. In relation to the employment situation, the 44.7% of the subjects were working away from home, 20.7% worked exceptionally from home (teleworking), and 34.5% were not working at the time of their participation in the studio. Most participants (70.1%) were spending COVID-19 confinement in a flat or apartment and the 28.8% was confined in a house. The 48.4% of the participants stated that they lived with children or youngsters under the age of 16 at the time of their participation in the study. Lastly, 9.1% indicated that they cohabited with people with disabilities.

### 2.2. Variables

This study aims to collect information on varied dimensions to assess the mental health and associated behaviours of the general population so as to assess the impact of this health crisis. Therefore, the dependent variable was psychological adjustment and as independent variables sociodemographic data, the presence of physical symptoms of COVID-19, participants’ health status, and history of possible contacts or exposure to COVID-19 were considered.

### 2.3. Instruments

The sociodemographic variables included were sex, age, marital status, education level, employment status, number of people living with, living with a child or adolescent, and living with a disabled person. The participants were asked about the prevalence of the most common symptoms of COVID-19 indicated by WHO over the past 14 days [41]: fever equal to or greater than 38 °C, cough, headache, myalgia, dizziness, diarrhoea, sore throat, coryza, chills, and difficulty in breathing. A self-developed questionnaire was designed including the symptoms as items and dichotomous responses (“Yes/No”). In relation to the state of health, the level of the participants’ current physical and mental health was assessed through dichotomous response questions (“Yes/No”) to the following items: suffering a chronic illness, having a disability, taking some medication, having been hospitalised in the last 14 days, and having been attended by some health service in the last 14 days. To this end, the process was based on Wang et al. approach [16]. An item with five response options was used to measure perceived health: very bad, bad, not so good, good, and very good. This indicator was initially proposed by Ilder and Benyamini [42] and used with small variants, in subsequent pandemic research [13,16,23,43]. It was assessed whether the person was quarantined due to having had a risk contact or COVID-19 infection, as well as whether they had had a COVID test. The contact history was evaluated by three questions with three answer categories (Yes, No, I don’t know), which evaluate direct/indirect contact with infected people or with people or materials suspected of being infected. A fourth taxonomic item (“Yes/No”) assessed cohabitating with people at risk of being infected.

Psychological adjustment was measured through the General Health Questionnaire (GHQ-12), a psychometric instrument widely used as a screening of non-psychotic psychiatric disorders [44]. It consists of 12 items with four answer options. Each item can get a score of 0 (if options 1 or 2 are chosen) or 1 (if options 3 and 4 are selected), getting from its sum a total score ranging from 0 to 12. This questionnaire developed by Goldberg has been translated and validated in many countries, presenting Cronbach’s alpha values from 0.82 to 0.86 [44] and demonstrating, in addition, a good reliability in its version for Spanish speakers with 0.86 and 0.76 in the Spanish population [45,46]; it has also been previously used in other SARS-like epidemics [23]. For this study, the overall score was used as a single factor whose reliability, estimated by Cronbach’s alpha, was of 0.851. The cut-off point set for the general population was 3, considering those subjects with scores greater than or equal to 3 more prone to potential psychiatric morbidity cases [47,48].

### 2.4. Procedure

The independent variables were assessed through a self-elaborated questionnaire. For the design of the questionnaire, a bibliographic review was carried out on the psychological effect that other epidemics, and their prevention measures, had had on the population in the past. With the accumulated evidence, a first version of the instrument was built and evaluated by a panel of experts made up of 10 health professionals: three doctors, four nurses, and three psychologists, two of which were specialists in clinical psychology. After the appropriate modifications were made, piloting was carried out with 57 participants, obtained through a sampling for convenience, all over 18 years of age and with a similar proportion of men and women in the sample (50.9% and 49.1%, respectively) and an average age of 41.87 (SD = 11.86). Most participants reported being married (56.1%) and having completed postgraduate studies, whether a master’s or doctoral degree (57.9%). They were all asked to complete the survey from different electronic devices. None of the participants expressed comprehension problems or doubts about what they were asked, nor were there any errors regarding the platform or design on the different devices (personal computer, Tablet or Smartphone) used by the participants.

Data were collected through the online data collection and survey platform Qualtrics^®^ XM. As a telematics application, the confinement measures decreed by the health alarm state did not affect data collection. The sampling method was through the “snowball” effect, initiated by sending the information through email lists to universities and professional colleges who were asked to facilitate their dissemination.

### 2.5. Ethical Considerations

The Helsinki Declaration [49] has been taken into consideration. Participation in the study was entirely voluntary, and the explicit permission of the participants was obtained through informed consent for the confidential use and processing of data, according to the current laws in force on the protection of personal data. Data were stored anonymously, with the assignment of a registration number so that it was not possible to identify the participants’ responses. The project was approved by the Research Ethics Committee of Huelva, belonging to the Andalusian Ministry of Health (PI 036/20).

### 2.6. Data Analysis

The analyses were performed using the SPSS 26.0 version statistical software (IBM, Armonk, NY, USA)-An initial descriptive analysis was performed by calculating the means and frequency of the variables. The presence or non-presence of psychological distress was studied in each of the independent variables. Subsequently, bivariate analyses were performed, including Chi-Squared Test and Student’s T-test for independent samples, depending on the type of variable. The size indexes of the Crammer’s V and Cohen’s d effect were also calculated with the following cut-off points: 0 to 0.19, negligible; 0.20 to 0.49, small; 0.50 to 0.79, medium; from 0.80 on, high [50]. Then, with the aim of studying the predictive ability for psychological distress of the different sets of variables, logistic regression analyses (controlled by sex and age) were carried out including variables with *p* value < 0.05. Thus, Model 1 included sociodemographic variables, and Model 2 was related to physical symptoms, Model 3 showed health-related variables, and Model 4 dealt with contact history. Finally, those variables that showed a predictive character in each of the models were included in a global model (Model 5). Odds ratios (ORs) were calculated with a 95% confidence interval.

## 3. Results

### 3.1. Psychological Distress

Table 1 details the mean scores and standard deviations of the answers provided by the subjects in each of the questions that make up the GHQ-12.

The results show that items 5 “Have you been constantly felt overwhelmed and tense?” (M = 2.88; SD = 0.88) and 7 “Have you been able to enjoy your normal activities every day?” (M = 2.81; SD = 0.87) were the ones with the highest score. On the contrary, the items that presented a lower score were the item 11 “Have you thought that you are a person who is worthless?” (M = 1.40; SD = 0.77) and item 10 “Have you lost self-confidence?” (M = 1.78; SD = 0.90). The average score obtained in the total of the 12 points scale was 4.99 (SD = 3.44). Establishing a cut-off point of 3 or more points, the results showed that a 72.0% of the 4180 study participants presented psychological distress.

### 3.2. Sociodemographic Variables and Psychological Distress

In the light of the sociodemographic variables (Table 2), the results showed statistically significant differences between both groups as for sex (χ² = 174.332, *p* ≤ 0.001, V = 0.204) and age (t = 9131, *p* ≤ 0.001, Cohen’s d = 0.337), though effect sizes were small. A greater presence of psychological distress was observed in women (79.6%) and in persons of lower middle age (M = 39.03, SD = 12.42) with respect to the group that did not present this psychic morbidity (M = 43.43, SD =14.51). Statistically significant differences were also found regarding the variable working situation (χ² = 63.139, *p* ≤ 0.001, V = 0.123) and in terms of living with children or youngsters under the age of 16 (χ² = 7.393, *p* = 0.007, V = 0.042). The highest percentage of psychological distress was observed among people who were working outside home (48.5%), and a low percentage of psychological distress was observed among people living with children or youngsters under the age of 16 (49.7%).

### 3.3. Physical Symptoms in the Past 14 Days and Psychological Distress

In relation to the presence of symptoms in the 14 days prior to the participation in the study (Table 3), more than half of the sample claimed to have had headache (53.3%); cough (30.6%), myalgia (29.3%), sore throat (27.2%), and coryza (20.1%). To a lesser extent, subjects reported having suffered from diarrhoea (17.1%), dizziness (13.0%), chills (12.2%), breathing difficulty (7.6%), and fever higher than 38 °C for at last one day (3.1%). On the other hand, according to the number of symptoms, the highest percentage (35.6%) stated that they had developed three or more symptoms in the 14 days prior to their participation in the study, followed by 23.4% of participants who had not developed any of these symptoms. Similar percentages were among those who reported having had a symptom (20.7%) and two symptoms (20.4%).

Statistically significant differences were observed between the presence of physical symptoms and psychological distress (*p* < 0.001 in all cases). Statistically significant differences were also found regarding the mean number of symptoms (t = −16.347, *p* ≤ 0.001, Cohen’s d = 0.510), with an average effect size. The group of subjects with psychological distress had a higher number of symptoms (M = 2.40, SD = 1.99), as compared to the group that did not present this psychic morbidity (M = 1.44, SD = 1.57).

### 3.4. Health-Related Variables and Psychological Distress

Based on health-related variables (Table 4), 29.8% of respondents reported suffering from some form of chronic disease. Among these subjects, the most commonly reported diseases were high blood pressure (29.0%) and chronic respiratory disease (25.3%), and to a lesser extent diabetes (8.3%), immunosuppression disease or situation (7.0%), metabolic syndrome (5.9%), chronic cardiovascular disease (5.0%), and active cancer (2.6%). Referring to the need for medical care, 0.6% of subjects reported having been hospitalised in the last 14 days, and 9.2% reported receiving healthcare at a health centre, clinic, or hospital. 4.9% of participants reported being quarantined for COVID-19 symptoms and 9.9% reported the diagnostic test (69.2% negative, 22.2% positive, and 8.7% do not know the result).

The variables related to the presence of psychological distress were the need for healthcare in a health centre, clinic, or hospital (χ² = 20.902, *p* < 0.001, V = 0.071), having been quarantined (χ² = 14.989, *p* < 0.001, V = 0.060), and having been done the diagnostic test (χ² = 27.174, *p* < 0.001, V = 0.081). For all of them, the size of the effect was negligible.

Lastly, and taking into account the subjects’ assessment of their perceived health in the last 14 days, the results also showed statistically significant differences between the two groups (t = 15.425, *p* ≤ 0.001, Cohen´s d = 0.540), with an average effect size. The group of subjects with psychological distress expressed a worse assessment of their health (M = 3.89, SD = 0.77), as compared to the group without psychological distress (M = 4.29, SD = 0.66).

### 3.5. Variables Related to Contact History in the Past 14 Days and Psychological Distress

In relation to contact history in the last 14 days (Table 5), 42.7% of participants reported having maintained or not knowing if they had maintained close contact with an individual with confirmed infection with COVID-19. 49.0% of respondents claimed to have had casual contact, and 59.7% said they had maintained or did not know if they had maintained contact with any person or material suspected of being infected with COVID-19. In relation to the presence of infected people in the participants’ immediate circle, 80.4% indicated not having a relative infected with the virus and 96.7% said they did not live with any confirmed infected family members. All contact history variables in the last 14 days showed a statistically significant relationship with the presence of psychological distress (*p* <.05 in all cases). However, the effect sizes were negligible.

### 3.6. Prediction of Psychological Distress

Logistic regression analyses have shown an adequate adjustment in general and an explained variance of 17.9% in the overall model, with correct classification percentages of each model around 73%, which has allowed to identify the predictive variables of psychological distress. Logistic regression models, controlled by sex and age, are displayed in Table 6.

Model 1 (sociodemographic variables) showed a predictive ability of 9.8% (χ² = 292.808, *p* < 0.001). The result of the Hosmer-Lemeshow test indicated that this model did not present a good fit (χ² = 22.806, *p* = 0.004). Sex, specifically female (OR = 2.316, 95% CI = (1.991, 2.694)), age (OR = 0.977, 95% CI = (0.72, 0.982)), and employment situation were predictive, correctly classifying 72.8% of subjects with sensitivity and specificity parameters of 96.4% and 11.9%, respectively.

With Model 2, regarding physical symptoms, the variance value explained amounted to 12.6% (χ² = 380.970, *p* < 0.001). Those participants who had a higher number of symptoms in the 14 days prior to their participation in the study (OR = 1.301, 95% CI = (1.245, 1.360)) were more likely to present psychological distress. This model correctly classified 73.0% of participants (sensitivity 95.0% and specificity 16.5%).

Model 3, which includes health-related variables, had a predictive capacity of 14.8% (χ² = 448.018, *p* < 0.001), slightly higher than the previous model. This model provided sensitivity and specificity values of 95.1% and 19.4%, correctly classified to 74.0% of the sample. However, it did not present a good fit (Hosmer-Lemeshow Chi-squared value =26.294, *p* < 0.01). Participants with a higher score in self-rated health (OR = 0.474, 95% CI = (0.424, 0.530)) were less likely to present psychological distress. However, those subjects who had recently been diagnosed with COVID-19 were 1.365 times more likely to have psychological distress (95% CI = 1.014, 1.838).

The contact history variables are included in Model 4, which provided an explained variance rate of 11.1% (χ² = 333.388, *p* < 0.001). Having had a close contact with an individual with confirmed infection with COVID-19 (OR = 1.391, 95% CI = (1.137, 1.701)), as well as having had any contact with any person or material suspected of being infected (OR = 1.415, 95% CI = (1.176, 1.702)) had predictive ability, correctly classifying 72.9% of the participants (95.3% sensitivity and 15.3% specificity).

Finally, Model 5 (Global Model), which included the variables that had a predictive character in the previous models, presented a predictive ability of 17.9%, correctly classifying 74.5% of the participants (93.5% sensitivity and 25.7% specificity). The variables that showed the greater weight, with ORs greater than 1, were sex (OR = 1.952, 95% CI = (1.667, 2.286)), number of symptoms presented in the last 14 days (OR = 1.130, 95% CI = (1.074, 1.190)), having had close contact with an individual with confirmed infection with COVID-19 (OR = 1.241, 95% CI = (1.026, 1.500)), and having had contact with any person or material suspected of being infected (OR = 1.258, 95% CI = (1.052, 1.503)). Other predictive variables with ORs less than 1 were age, employment status, and self-rated health.

## 4. Discussion

In this study, various sociodemographic variables, variables related to the presence of physical symptoms, and other health-related ones have been identified as predictors of the presence of psychological distress symptoms among the Spanish population during a period of health alert due to the COVID-19 epidemic. In Spain, during the initial moments of confinement, 72.0% of the study participants showed risk of psychiatric morbidity (or distress). This figure is much higher than the ones found in previous studies carried out on the Spanish population, that placed psychiatric morbidity at 18.0% [51] or 19.1% [52], not having subsequent data [42]. Specific studies on the psychological impact during epidemics place the prevalence of psychological distress between 22.9% and 56.7% [20,21,22,23,24,53]. In our study, the highest percentage level of psychological vulnerability during an epidemic can be found. These high results may be due to the fact that the COVID-19 pandemic in our country has affected the Spanish population in a more serious way than previous pandemics and the feeling of alarm is greater.

As for the role that sex may play in relation to psychological vulnerability in epidemic situations, some studies have found that being male was associated with greater distress during the recovery period of SARS [20] but, in most studies, females were associated with greater vulnerability. Women are found to suffer greater distress during the H_1_N_1_ influenza outbreak [28] or during equine influenza [21], and a longitudinal study on the impact of the SARS outbreak in Hong Kong [54] found that women were more likely to suffer anxiety. One of the first studies conducted during the COVID-19 epidemic identified an increased risk of anxiety, depression, and stress among women [16]. As for the general indicators of mental health in Spain, being a woman is associated with greater vulnerability [51]. Our results are in line with those found in most studies, showing that women present significantly higher levels of distress (with low size effect), and this can therefore be understood as an individual risk factor in the face of the impact of the COVID-19 epidemic.

The results show that, although weakly, younger people are at higher risk of suffering higher levels of distress. These data are consistent with those from previous studies in epidemic states, and in which being younger was associated with an increased risk of distress [21] or increased psychiatric morbidity [53]. However, a study similar to the present one conducted at the beginning of the COVID-19 quarantine identified an increased risk of psychological distress among people over 60 years of age [33]. Mental health indicators in Spain show that psychiatric morbidity increases with age [49]. Our data indicate that the youngest part of the study population is the one with the highest psychiatric morbidity. This result can be understood in line with Sim [53] and Taylor [21], due to the relationship they establish regarding differences in coping styles. Thus, youngest adults are less resilient in the face of adversity and also less able to understand that it is an extreme situation that implies radical and sudden changes in the lives of people, and which are not the result of an individual decision.

As for the relationships found between the degree of distress and living with children during confinement, the data showed that people with children have a greater psychological vulnerability. However, the effect size was negligible. This result coincides with those by Taylor’s study [19] which suggests that people with a child are more likely to have psychological distress, explaining that those who have a child are usually younger adults and hence the association with the greatest risk. On the other hand, as Brooks [10] and Naushad [29] state, there is no link between having children and any psychological impact in their reviews. However, Mazza et al., in their recent study, conducted during the COVID-19 crisis in Italy, identified an association between having no children and a higher level of depression [36].

In relation to the employment situation, most previous studies have analysed the role of economic income and its changes as a result of labour measures taken during an epidemic. Thus, reduced or low level of economic income was consistently related to an increased risk of psychological impact [10,13,21]. General indicators of mental health in Spain show that low levels or lack of economic income, as well as lack of employment, are associated with lower mental health [51]. Our data, however, indicated, weakly, that in the pandemic situation by COVID-19, those who have to work away from home had a higher level of distress. This outcome can be related with a higher risk of contagion and concern for all its consequences, as they can spread the disease to the family and due to the high degree of uncertainty about the disease. In this line, Mihashi’s study [20] shows how the perception of risk is associated with psychiatric morbidity during and after recovery of SARS. On the other hand, the results obtained by Jahanshahi et al. on the effects of COVID-19 quarantine in Iran suggest that participants who had to stop working because of the pandemic had more psychological distress than those who worked from home or at their workplace [34].

Quarantine for at least 14 days is associated with increased anxiety and anger [14], as well as with increased symptoms of post-traumatic stress disorder [13,19,30]. Our data showed that quarantine was associated with increased psychiatric morbidity (negligible size effect). In the line posed by Hawryluck et al. [13], being quarantined can be interpreted by these people like trauma or personal assault.

Our study coincides with previous ones associating perceived low health with a higher level of stress and psychological impact in general [16,53]. We have also observed, with an average size effect, that a worse perception of health was linked to increased psychiatric vulnerability.

The presence of COVID-19 symptoms was also related to the level of distress, so the presence of some symptoms can be considered a factor associated with increased psychological morbidity. The study on the psychological impact of COVID-19 conducted by Wang [16] identified that myalgia, dizziness, chills, sore throat, and having a cold were associated with a greater psychological impact of the outbreak. Additionally, the presence of COVID-19 symptoms was associated with higher levels of stress, anxiety, and depression [31]. Similarly, during an outbreak of SARS, the presence of symptoms such as fever was linked to the risk of higher distress, which can be understood on the basis that the onset of symptoms can reinforce the sense of vulnerability and threat of infection [53]. The presence of MERS symptoms was related to an increase in anger scores [14].

Also, testing and history of contact with infected people or objects were related, with negligible size effect, to increased psychiatric morbidity. No data from previous studies reveal the role that testing or diagnostic tests may play on the effects of psychological morbidity, but there are studies that indicate that the presence of risk contact may be a predictor of acute stress disorder [10] or post-traumatic stress disorder [19], having found a relationship between anxiety and having had contact with materials suspected of being infected [16]. Our result can answer to what Wu [19] raised by showing that protective measures are often relaxed with close people such as family and friends. However, knowing that contact has been made with a person who has subsequently become ill increases the feeling of danger, similar to the risk of contracting the illness, also increasing psychological vulnerability.

Among the limitations of the study, as it is cross-sectional observational design which only informs of the perception at the time it was performed, it does not allow to establish cause-and-effect relationships but, on the contrary, it does provide with very valuable and difficult to obtain information about how the problem generated by the pandemic is lived just at the time of further escalation of the contagion curve, this being the largest contribution of the article. The sample collection was not randomised and the ratio by sex was asymmetrical and does not correspond to the distribution of the Spanish population. These factors were compensated with a large sample and representation from all provinces and autonomous cities, having taken into account the variable sex in the analysis. Comparing these data with those from other epidemics is difficult because the measures established in confinement or isolation are highly variable, even in different geographical areas within the same pandemic, variables that, as seen in this study, have a great influence in the development of psychological distress. It may be interesting to make other cuts of the study at a more advanced stage of the pandemic and at the end of the pandemic in order to assess its evolution.

## 5. Conclusions

The study, conducted during the health alert decree with confinement measures at home except for essential activities, and initiated at the beginning of growth of the contagion curve, shows that a high percentage (72%) of participants had psychological distress, being this percentage higher among women (79.6%). People who work outside home in essential activities are more likely to suffer psychological distress, and those who lived with children or under-16 youngsters were less likely to show this distress. The most common symptoms in the last 14 days were headache, cough, myalgia, sore throat, and rhinitis; three or more symptoms are more commonly found. Although one out of three participants had a chronic disease, only 9.2% had required health care and less than one percent (0.6%) required hospital care.

A high percentage (42.7%) claimed to have had contact or not knowing whether they had had contact with any infected person or material. However, a vast majority claimed not having had any infected family member (80.4%) and not living with any infected family member (96.7%). An association was found between psychological distress and a poor assessment of health. Among the variables that predict psychological distress are, therefore: being female, age, employment situation, number of symptoms, perception of poor health, having been in close contact with an infected person, as well as having been in contact with people or material suspected of being infected.

These results should be explored in depth and considered for awareness-raising and information programmes during pandemics or other crisis situations, as they could enrich prevention interventions in public health and, in particular, in mental health. The information provided by the present study can help to design interventions for the psychological and emotional recovery of the population after the pandemic. It can also help in the design of mental health prevention programs aimed to protect the population from psychological distress in case of future pandemics.

## Figures and Tables

**Table 1:** General Health Questionnaire GHQ-12 (N = 4180).

Items	M (SD)
1. Have you been able to concentrate well on what you were doing?	2.68 (0.72)
2. Have your worries made you lose a lot of sleep?	2.68 (0.97)
3. Have you felt that you are playing a useful role in life?	2.02 (0.85)
4. Have you felt capable of making decisions?	2.12 (0.66)
5. Have you felt constantly overwhelmed and stressed?	2.88 (0.88)
6. Have you had the feeling that you cannot overcome your difficulties?	2.22 (0.92)
7. Have you been able to enjoy your normal daily activities?	2.81 (0.87)
8. Have you been able to adequately cope with your problems?	2.33 (0.65)
9. Have you felt unhappy or depressed?	2.55 (0.98)
10. Have you lost confidence in yourself?	1.78 (0.90)
11. Have you thought that you are a person worthless?	1.40 (0.77)
12. Do you feel reasonably happy considering all the circumstances?	2.28 (0.74)
**Scale total (over 12 points)**	4.99 (3.34)
**Presence of psychological distress (cut point ≥ 3)**	(%)
Yes	72.0
No	28.0

**Table 2:** Association between sociodemographic variables and psychological distress during the COVID-19 pandemic (N = 4180).

	*N* (%)	Psychological Distress		*χ²/t*	*p*	Effect Size
		No (*N* = 1171)	Yes (*N* = 3009)			
**Sex**						
Male	1088 (26.0)	40.4	20.4	174.332	<0.001	0.204
Female	3092 (74.0)	59.6	79.6			
**Age (mean (SD))**	40.26 (13.18)	43.43 (14.51)	39.03 (12.42)	9.131	<0.001	0.337
**Marital Status**						
Single	1419 (33.9)	31.9	34.7	3.833	0.280	0.030
Married or living as a couple	2416 (57.8)	59.2	57.3			
Separated/Divorced	296 (7.1)	7.4	6.9			
Widowed	49 (1.2)	1.5	1.1			
**Educational level**						
Primary education	57 (1.4)	1.2	1.4	5.654	.130	0.037
Lower secondary education	65 (1.6)	2.0	1.4			
Upper secondary education	838 (20.0)	21.9	19.4			
University or higher	3212 (76.9)	74.9	77.8			
**Employment status**						
Working away from home	1869 (44.7)	35.1	48.5	63.139	<0.001	0.123
Working from home	867 (20.7)	25.7	18.8			
Not working	1444 (34.5)	39.2	32.7			
**Type of dwelling**						
Flat or Apartment	2929 (70.1)	68.6	70.7	1.772	0.412	0.021
House	1205 (28.8)	30.3	28.2			
Others (i.e.: residence, hotel, etc.)	46 (1.1)	1.1	1.1			
**Living with children or under-16 youngsters**						
No	2180 (51.6)	55.0	50.3	7.393	0.007	0.042
Yes	2022 (48.4)	45.0	49.7			
**Living with disabled people**						
No	3798 (90.9)	90.1	91.2	1.153	0.283	0.017
Yes	382 (9.1)	9.9	8.8			

**Table 3:** Association between physical symptoms in the past 14 days and psychological distress during the COVID-19 pandemic (N = 4180).

	*N* (%)	Psychological Distress		χ*²/t*	*p*	Effect Size
		No (*N* = 1171)	Yes (*N* = 3009)			
**Fever (> 38 °C for at least 1 day)**						
No	4051 (96.9)	98.4	96.3	11.650	0.001	0.053
Yes	129 (3.1)	1.6	3.7			
**Cough**						
No	2901 (69.4)	75.7	67.0	30.018	<0.001	0.085
Yes	1279 (30.6)	24.3	33.0			
**Headache**						
No	1954 (46.7)	61.0	41.2	132.266	<0.001	0.178
Yes	2226 (53.3)	39.0	58.8			
**Myalgia**						
No	2955 (70.7)	80.6	66.8	77.284	<0.001	0.136
Yes	1225 (29.3)	19.4	33.2			
**Dizziness**						
No	3635 (87.0)	93.6	84.4	63.132	<0.001	0.123
Yes	545 (13.0)	6.4	15.6			
**Diarrhoea**						
No	3464 (82.9)	89.3	80.4	47.742	<0.001	0.107
Yes	716 (17.1)	10.7	19.6			
Sore **throat**						
No	3041 (72.8)	82.4	69.0	76.526	<0.001	0.135
Yes	1139 (27.2)	17.6	31.0			
**Coryza**						
No	3340 (79.9)	85.5	77.7	31.523	<0.001	0.087
Yes	840 (20.1)	14.5	22.3			
**Chills**						
No	3671 (87.8)	93.8	85.5	53.725	<0.001	0.113
Yes	509 (12.2)	6.2	14.5			
**Breathing difficulty**						
No	3863 (92.4)	95.4	91.3	20.505	<0.001	0.070
Yes	317 (7.6)	4.6	8.7			
**Number of symptoms (mean (SD))**	2.13 (1.93)	1.44 (1.57)	2.40 (1.99)	−16.347	<0.001	0.510

**Table 4:** Association between health-related variables and psychological distress during the COVID-19 pandemic (N = 4180).

	*N* (%)	Psychological Distress		*χ²/t*	*p*	Effect Size
		No (*N* = 1171)	Yes (*N* = 3009)			
**Chronic diseases**						
No	2935 (70.2)	69.9	70.3	0.059	0.808	0.004
Yes	1245 (29.8)	30.1	29.7			
**Currently taking any medication**						
No	2583 (61.8)	61.8	61.8	0.001	0.978	0.000
Yes	1597 (38.2)	38.2	38.2			
**Recent hospitalisation in the past 14 days**						
No	4154 (99.4)	99.6	99.3	1.001	0.317	0.015
Yes	26 (0.6)	0.4	0.7			
**Health care in a health centre, clinic or hospital in the past 14 days**						
No	3797 (90.8)	94.1	89.6	20.902	<0.001	0.071
Yes	383 (9.2)	5.9	10.4			
Self-rated health in the past 14 days *	4.01 (0.76)	4.29 (0.66)	3.89 (0.77)	15.425	<0.001	0.540
**Recent quarantine in the past 14 days for having symptoms**						
No	3838 (91.8)	94.4	90.8	14.989	<0.001	0.060
Yes	203 (4.9)	5.6	9.2			
**Recent testing for COVID-19 in the past 14 days**						
No	3765 (90.1)	93.9	88.6	27.174	<0.001	0.081
Yes	415 (9.9)	6.1	11.4			

*Note: Likert-type scale from 0 (very bad) to 5 (very good).

**Table 5:** Association between contact history variables in the past 14 days and psychological distress during the COVID-19 pandemic (n = 4180).

	*N* (%)	Psychological Distress		*χ²/t*	*p*	Effect Size
		No (*N* = 1171)	Yes (*N* = 3009)			
**Close contact with an individual with confirmed infection with COVID-19**						
No	2395 (57.3)	69.6	52.5	100.617	<0.001	0.155
Yes or does not know	1785 (42.7)	30.4	47.5			
**Casual contact with an individual with confirmed infection with COVID-19**						
No	2130 (51.0)	62.3	46.5	84.341	<0.001	0.142
Yes or does not know	2050 (49.0)	37.7	53.5			
**Contact with any person or material suspicious of being infected with COVID-19**						
No	1683 (40.3)	52.5	35.5	101.593	<0.001	0.156
Yes or does not know	2497 (59.7)	47.5	64.5			
**Any infected family member**						
No	3362 (80.4)	83.9	79.1	12.154	<0.001	0.054
Yes or does not know	818 (19.6)	16.1	20.9			
**Living with an infected family member**						
No	4043 (96.7)	97.8	96.3	5.735	0.017	0.037
Yes	137 (3.3)	2.2	3.7			

**Table 6:** Logistic regression models on psychological distress by set variables.

Variables		Model 1 OR (95% CI) Sociodemographic Variables	Model 2 OR (95% CI) Physical Symptoms	Model 3 OR (95% CI) Health-Related Variables	Model 4 OR (95% CI) Contact History	Model 5 OR (95% CI) Global Model
	**Regression models**	R² = 0.098 (96.4/11.9%)	R² = 0.126 (95.0/16.5%)	R² = 0.148 (95.1/19.4%)	R² = 0.111 (95.3/15.3%)	R² = 0.179 (93.5/25.7%)
**Sociodemographic**	Sex (ref. male)	2.316 ** (1.991, 2.694)	2.082 ** (1.786, 2.427)	2.146 ** (1.839, 2.504)	2.213 ** (1.901, 2.577)	1.952 ** (1.667, 2.286)
	Age	0.977 ** (0.72, 0.982)	0.983 ** (0.977, 0.988)	0.977 ** (0.972, 0.982)	0.978 ** (0.973, 0.983)	0.976 ** (0.971, 0.982)
	Employment status (ref. working away home)					
	Working from home	0.569 ** (0.474, 0.683)	NA	NA	NA	0.706 * (0.579, 0.861)
	Not working	0.561 ** (0.476, 0.661)	NA	NA	NA	0.611 ** (0.509, 0.733)
	Living with children under the age of 16	1.152 (0.999, 1.328)	NA	NA	NA	NA
**Physical symptoms**	Number of symptoms	NA	1.301 ** (1.245, 1.360)	NA	NA	1.130 ** (1.074, 1.190)
**Health-related**	Health care in a health centre, clinic or hospital	NA	NA	1.169 (0.873, 1.564)	NA	NA
	Self-rated health	NA	NA	0.474 ** (0.424, 0.530)	NA	0.561 ** (0.495, 0.636)
	Recent quarantine for having symptoms	NA	NA	0.907 (0.662, 1.241)	NA	NA
	Recent testing for COVID-19	NA	NA	1.365 * (1.014, 1.838)	NA	0.908 (0.674, 1.223)
**Contact history**	Close contact with an individual with confirmed infection with COVID-19	NA	NA	NA	1.391 ** (1.137, 1.701)	1.241 * (1.026, 1.500)
	Casual contact with an individual with confirmed infection with COVID-19	NA	NA	NA	1.198 (0.982, 1.463)	NA
	Contact with any person or material suspicious of being infected	NA	NA	NA	1.415 ** (1.176, 1.702)	1.258 * (1.052, 1.503)
	Any confirmed family member	NA	NA	NA	1.080 (0.882, 1.322)	NA
	Living with a confirmed family member	NA	NA	NA	1.114 (0.692, 1.795)	NA

* *p* < 0.05; **p < 0.01; NA: not applicable; R² = Model explained variance (sensitivity / specificity); OR (95% CI): Odds Ratio (Confidence Interval at the 95% level).
